# Molecular and Immune Characteristics for Lung Adenocarcinoma Patients With ERLIN2 Overexpression

**DOI:** 10.3389/fimmu.2020.568440

**Published:** 2020-12-07

**Authors:** Yifei Liu, Pengfei Xie, Daishang Jiang, Jian Liu, Jianguo Zhang, Tingting Bian, Jiahai Shi

**Affiliations:** ^1^ Department of Pathology, Affiliated Hospital of Nantong University, Nantong, China; ^2^ Medical School of Nantong University, Nantong, China; ^3^ Department of Thoracic Surgery, Affiliated Tumor Hospital of Nantong University, Nantong, China; ^4^ Department of Emergency Medicine, Affiliated Hospital of Nantong University, Nantong, China; ^5^ Department of Chemotherapy, Affiliated Hospital of Nantong University, Nantong, China; ^6^ Departments of Cardio-Thoracic Surgery, Affiliated Hospital of Nantong University, Nantong, China

**Keywords:** ERLIN2, lung adenocarcinoma, prognosis, survival, tumor-infiltrating immune cells

## Abstract

**Background:**

Endoplasmic reticulum lipid raft-associated protein 2 (ERLIN2) is protein contained in the membrane of the endoplasmic reticulum. In lung adenocarcinoma (LUAD), the molecular function of ERLIN2 and the correlation between ERLIN2 and tumor-infiltrating immune cells have been unclear. The aim of our study was to determine the role of ERLIN2 in LUAD development to provide a better understanding of the molecular pathogenesis of this disease and identify new therapeutic targets for its treatment.

**Methods:**

Immunohistochemistry, Western blotting, and real-time quantitative polymerase chain reaction were used to detect protein and mRNA levels of ERLIN2 in LUAD and adjacent normal tissues. Using the A549, H1299 cell line, ERLIN2-short hairpin RNA was applied to silence ERLIN2 to determine its role in LUAD cell proliferation and invasion. Based on mRNA expression of ERLIN2 from the Cancer Genome Atlas (TCGA) database, we identified ERLIN2-related protein-coding genes and analyzed the Kyoto Encyclopedia of Genes and Genomes pathway to explore its potential biological functions and determined the correlation between ERLIN2 and tumor-infiltrating immune cells.

**Results:**

ERLIN2 was abnormally expressed in a variety of tumor tissues and is highly expressed in LUAD. This overexpression was associated with histological grade (P = 0.044), TNM stage (P = 0.01), and lymph node metastasis (P = 0.038). Patient overall survival was poorer with ERLIN2 overexpression. Downregulation of ERLIN2 inhibited LUAD cell proliferation and invasion in vitro. Based on mRNA expression of ERLIN2 from the TCGA database, 13 ERLIN2-related genes and 10 pathways were identified and showed a correlation between ERLIN2 and naive B cells and neutrophils.

**Conclusion:**

ERLIN2 could serve as a potential diagnostic and prognostic biomarker for LUAD and has demonstrated to be correlated with immune infiltrates, which suggests that it may represent a new therapeutic target for LUAD.

## Introduction

Lung cancer is one of the main cancer-related deaths worldwide, accounting for ~20% of all cancer deaths (1). Lung adenocarcinoma (LUAD) is the most important subtype of lung cancer and usually metastasizes, which leads to a poor prognosis for the patient (2). Although the existing treatment methods have made some progress, the 5-year survival rate remains only 10–20% (3), which is important when identifying the current therapeutic restrictions associated with the disease. The mechanisms of LUAD tumorigenesis remain unclear; therefore, it is urgent that we identify any biomarkers for the diagnosis of this disease.

Endoplasmic reticulum lipid raft-associated protein 2 (ERLIN2), also known as stomatin/prohibitin/flotillin/HflK/C (SPFH2) or C8ORF2, is a protein within the membrane of the endoplasmic reticulum (ER) that contains an evolutionarily conserved SPFH domain (4). In recent years, ERLIN2 has been considered to be a new medium related to ER degradation by binding to activated inositol triphosphate receptors (IP3Rs) and other ER-related degradation substrates, leading to polyubiquitination and their subsequent degradation (5). ERLIN2 can also interact with ER-resident protein from *insulin-induced gene 1* to regulate the activation of sterol regulatory element binding protein 1c without acting as an ER degradation medium (6). Through this regulating mechanism, ERLIN2 helps the cells maintain high levels of cytoplasmic lipids and gain a growth advantage during tumorigenic stress. ERLIN2 gene mutation has been found to be related to motor neuron diseases in children (7). In the research, ERLIN2 has been reported only in breast cancer to indicate its effects on the cell-cycle processes of breast cancer cells (8).

As far as we know, there has been no study on the role of ERLIN2 in LUAD carcinogenesis; therefore, the aim of our study was to explore the expression of ERLIN2 in LUAD samples and analyze the correlation between ERLIN2 expression and certain clinical parameters, as well as the prognosis for LUAD patients.

## Materials and Methods

### Lung Adenocarcinoma (LUAD) Clinical Samples and Immunohistochemistry Assay

At the Affiliated Hospital of Nantong University in China, 284 pairs of LUAD and adjacent normal tissues were treated. All patients were treated by surgical resection between 2007 and 2011. All clinical data on the patients were carefully recorded after the diagnosis of LUAD by two pathologists. The pathological stage was determined according to the *8th Edition of the TNM Classification for Lung Cance*r (9). The follow-up was completed by June 30, 2014, and the median follow-up duration was 52 months. All experiments involving patient specimens were approved by the Ethics Committee of the Affiliated Hospital of Nantong University, China.

An immunohistochemistry (IHC) assay was conducted as previously described (10). Briefly, the LUAD samples were deparaffinized and rehydrated. The primary antibodies were those against ERLIN2 (1:100 dilution; ab129207; abcam). The scoring criteria for IHC staining were based on the intensity of the stain and the percentage of immunoreactive cells, as previously described (10).

### Analyses of Western Blotting and Real-Time Quantitative Polymerase Chain Reaction

Western blotting analyses were conducted as previously described (11) using 50 μg protein samples from fresh tissues. The primary antibodies were ERLIN2 (1:2,000 dilution; ab129207; abcam) and *β*-actin (1:10,000 dilution; 66009-1; proteintech). RNA from tumor tissues was extracted using TRIzol Reagent (Invitrogen), and the cDNA was obtained through reverse transcription using a PrimeScript™ RT Reagent Kit (TaKaRa, Shiga, Japan). Real-time quantitative polymerase chain reaction (qPCR) was conducted in triplicate for each cell sample using a SYBR Premix Ex Taq II Reagent Kit (TaKaRa).

The primer sequences for the target genes were as follows: ERLIN2 forward 5′-TCCACCACGAACTGAACCAG-3′,reverse5′-AACAGCTCAATGTAGACCTCTTG-3′; GAPDH forward 5′-TGACTTCAACAGCGACACCCA-3′, reverse 5′-CACCCTGTTGCTGTAGCCAAA-3′.

### Lung Adenocarcinoma (LUAD) Cell Lines and Cell Culture

The A549, H1299 human LUAD cell line was purchased from the American Type Culture Collection (Manassas, VA, USA). The A549, H1299 cells were cultured in swell Park Memorial Institute-1640 medium (Thermo Fisher Scientific, Inc., Waltham, MA, USA) containing 10% fetal bovine serum (Haoyang Biological Manufacture Co. Ltd., Tianjin, China) and 100 units penicillin–streptomycin in a humidified atmosphere with 5% CO_2_ at 37°C.

### Construction of Plasmids and Transfection

A549, H1299 cells were transfected with plasmids encoding ERLIN2, or short-hairpin (sh)RNA against ERLIN2, along with vector control. The shRNA targeting sequence for ERLIN2 was GGGTAACAAAGCCCAACATAC. The cDNA encoding full-length human ERLIN2 was cloned into a PCDH vector. The expression constructs were confirmed by DNA sequencing. The transfection process was as described before (10).

### Cell Viability Assays, 5-Ethynyl-2′-Deoxyuridine Staining, Cell Wound Healing Assay, and Transwell Assay

Cell viability was measured using 3-(4,5-dimethylthiazol-2-yl)-2,5-diphenyltetrazolium bromide (Sigma-Aldrich) according to the manufacturer’s instructions. Cell growth was also evaluated using the 5-ethynyl-2′-deoxyuridine kit (RiboBio, Science City, China) according to the manufacturer’s instructions. Cells were cultured to full confluence in a six-cell plate, after which a micropipette tip was used to scratch the surface. The cells were washed with phosphate-buffered saline and cultured in serum-free medium. The scratches were then photographed at 0, 24, and 48 h, and cell migration was compared by measuring the gap size in each field. A549, H1299 cell invasion assay was using a Transwell system (Corning, Tewksbury, MA) based on previously described methods (12).

### Flow Cytometry Analysis

The BD Fluorescence-activated cell sorting (FACS) Calibur flow cytometry system (Becton Dickinson, Franklin Lakes, NJ, USA) was used to detect cell cycle distribution. A549, H1299 cells were harvested and fixed with 70% ice-cold ethanol. After treating with RNaseA, the cells were stained with propidium iodide (PI) for 30 min.

### Gene Set Enrichment Analysis

In the Cancer Genome Atlas (TCGA)–Persons at Risk (PARD) database, 535 LUAD cases were divided into two expression-level groups according to the median expression value of ERLIN2. A gene set enrichment analysis was then conducted to detect the gene sets that were enriched in the gene rank in the two groups for identifying a potential hallmark of LUAD. For each analysis, 1,000 repetitions of gene set permutations were completed. The phenotype label put forth was the expression level of ERLIN2. In addition, we used the nominal p-value and normalized enrichment score to sort the enriched pathways in each phenotype (13). Gene sets with a discovery rate (FDR) <0.05 were considered to be significantly enriched.

### Immune Infiltrate Analysis

To assess the relative variations of gene expression among sets in the samples, we used the deconvolution algorithm CIBERSORT based on gene expression (http://cibersort.stanford.edu/) (14) to measure the immune response of 21 tumor infiltrating immune cells (TIICs), evaluate their association with ERLIN2 expression in LUAD, and determine any correlation among TIICs. We used standard annotation files to establish gene expression datasets and the default signature matrix at 1,000 permutations. Using the Monte Carlo method, CIBERSORT approximated a p-value for deconvolution to determine the levels of confidence in the results. To analyze the influence of ERLIN2 on the microenvironment of the immune system, 338 tumor samples were used and classified into two groups. To determine the types of lymphocytes affected by ERLIN2, p-value <0.05 was set as significant.

The Tumor and Immune System Interactions Database (TISIDB) provides a user-friendly web portal (http://cis.hku.hk/TISIDB) that allows users to explore the function of a gene of interest and its role in tumor–immune interactions through high-throughput data analysis and literature mining (15).

### Statistical Analyses

The data acquired from TCGA were merged and analyzed using R 3.5.3. The correlations between the clinical information and ERLIN2 expression were analyzed using logistic regression. The multivariate Cox analysis was used to evaluate the influence of ERLIN2 expression on survival. All of the experiments were repeated at least three times. Differences between groups were determined using Student’s t-test. P-value <0.05 indicated statistical significance.

## Results

### The Levels of Endoplasmic Reticulum Lipid Raft-Associated Protein 2 (ERLIN2) mRNA in Lung Adenocarcinoma (LUAD) and Other Cancers

**Figure 1:**
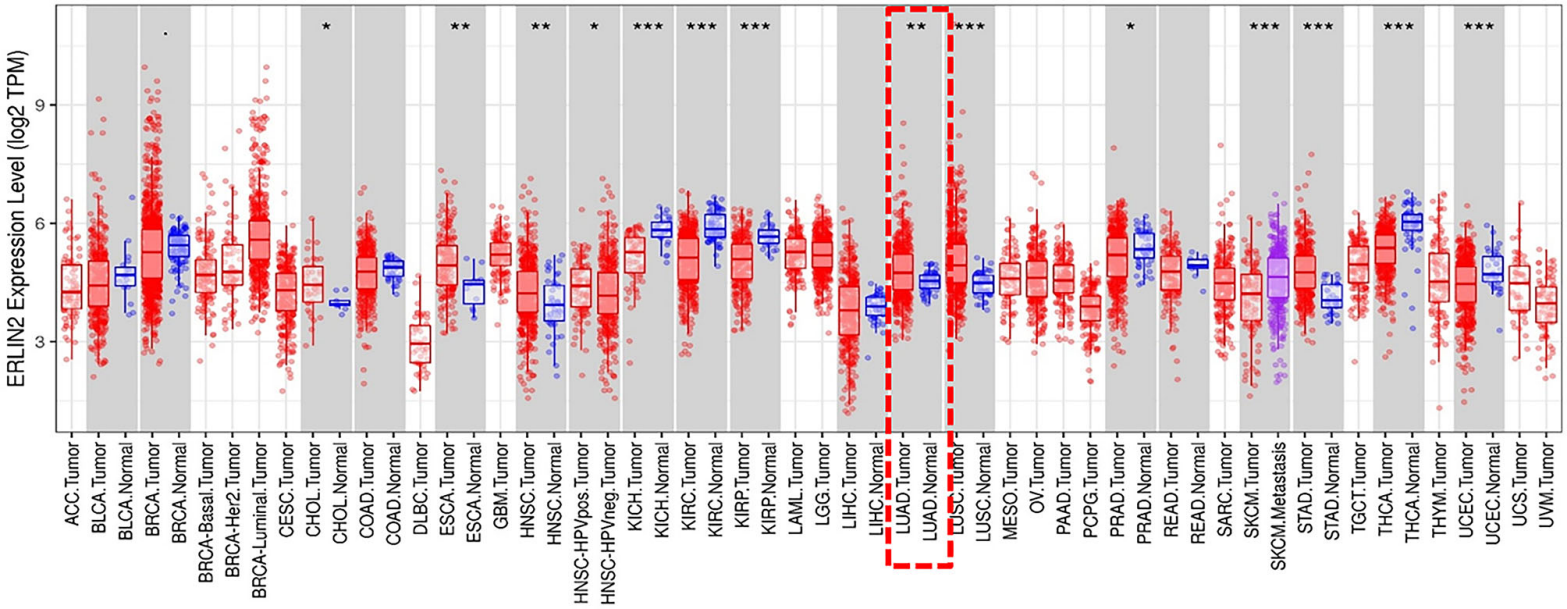
The level of ERLIN2 expression in different tumor types from the TCGA database in TIMER. Note: ^*^P < 0.05, ^**^P < 0.01, ^***^P < 0.001.

The analysis of TCGA RNA-seq data using the TIMER database (http://cistrome.org/TIMER/) showed that ERLIN2 mRNA expression was significantly higher in esophageal carcinoma, head and neck cancer, lung adenocarcinoma, lung squamous cell carcinoma, and stomach adenocarcinoma and lower in kidney chromophobe, kidney renal clear cell carcinoma, kidney renal papillary carcinoma, thyroid carcinoma, and uterine corpus endometrial carcinoma tissues compared with that in adjacent normal tissues (**Figure 1** **)** (**Supplementary Figure 1**).

### Endoplasmic Reticulum Lipid Raft-Associated Protein 2 (ERLIN2) Predicts Poor Prognosis for Lung Adenocarcinoma (LUAD) Patients

**Figure 2:**
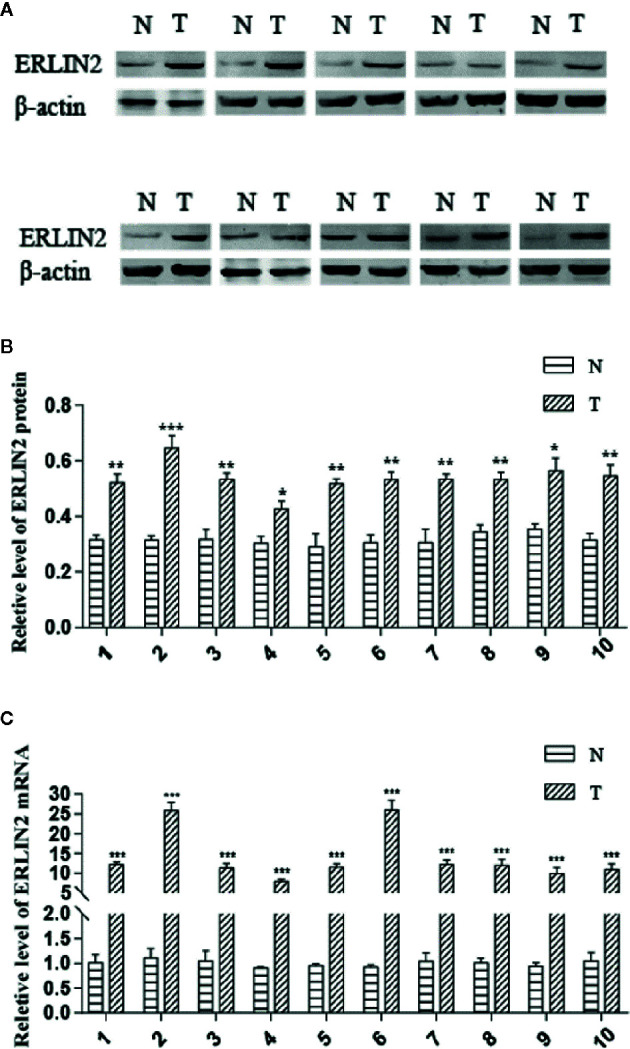
ERLIN2 level was significantly higher in LUAD tissues than in adjacent normal tissues. **(A, B)** ERLIN2 protein was determined by Western bolt and relative quantification analysis by normalizing to *β*-actin. **(C)** ERLIN2 mRNA was determined by qRT-PCR. All of the experiments were repeated at least three times. Note: ^*^P < 0.05, ^**^P < 0.01, ^***^P < 0.001.

**Figure 3:**
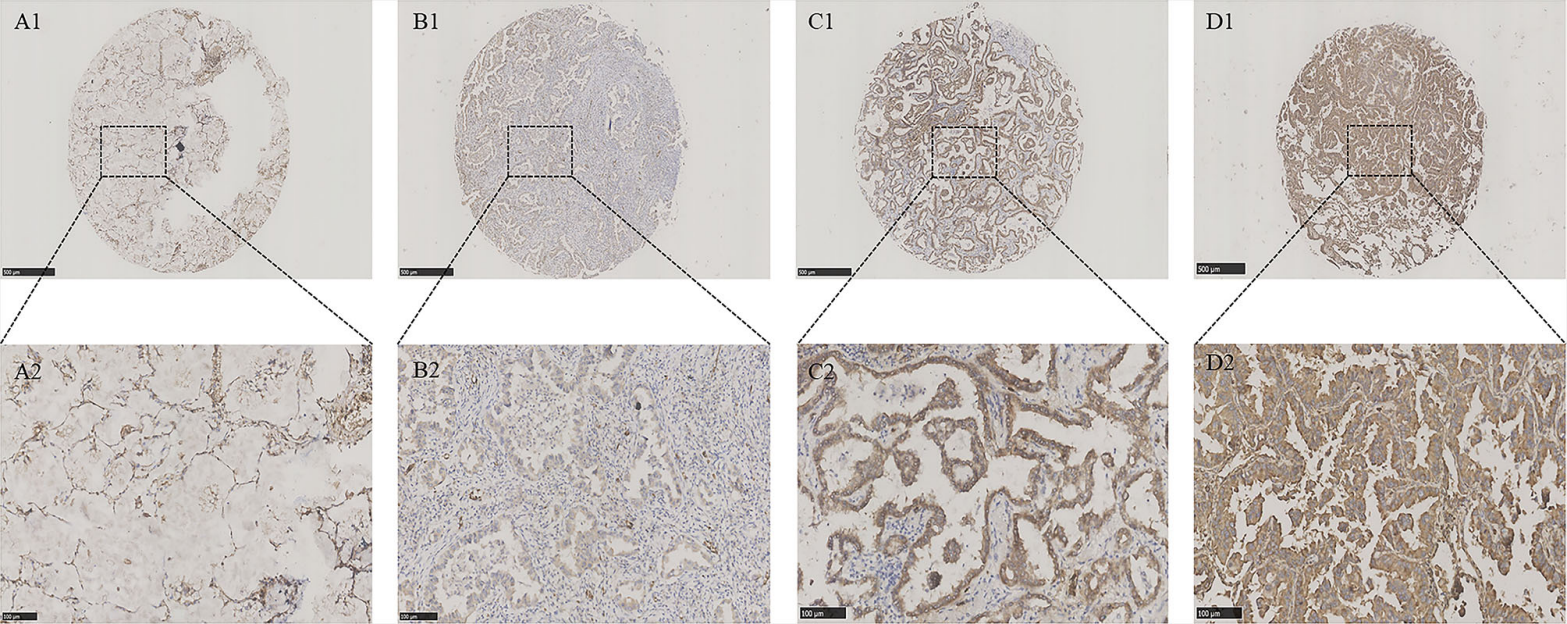
ERLIN2 protein was detected in LUAD tissues and normal lung tissues. ERLIN2 protein was determined by TMA-IHC. **(A1–A2)** Normal lung tissues, negative for ERLIN2 protein expression; **(B1–B2)** LUAD tissues, weak positive for ERLIN2 protein expression; **(C1–C2)** LUAD tissues, moderate positive for ERLIN2 protein expression; **(D1–D2)** LUAD tissues, strong positive for ERLIN2 protein expression. **A1, B1, C1** and **D1** are ×40 magnification (bar = 500 μm), **A2, B2, C2** and **D2** are ×200 magnification (bar = 100 μm). ERLIN2 protein was dyed brown particles in cell membranes.

**Figure 4:**
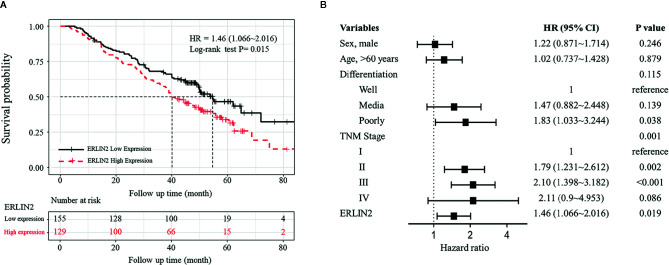
Kaplan–Meier analysis and multivariate Cox of ERLIN2 expression in LUAD. **(A)** ERLIN2 overexpression was negatively associated with the overall survival rate of patients (P = 0.015). **(B)** ERLIN2 expression is an independent prognostic factor in LUAD.

To explore the role of ERLIN2 in the development of LUAD, we first analyzed the expression levels of ERLIN2 in 10 pairs of fresh tumor tissues and matched normal tissues using Western blotting and qPCR. Our results showed that ERLIN2 was markedly overexpressed in the LUAD samples compared with that in the matched adjacent normal tissues (**Figure 2**). The results of IHC also revealed that ERLIN2 was overexpressed in LUAD compared with that in adjacent normal tissues (**Figure 3**). ERLIN2 was also highly expressed in 129 of 284 (45.4%) lung-tumor tissues. Moreover, the expression levels of ERLIN2 were correlated with histological grade (P = 0.044), TNM stage (P = 0.01), and lymph node metastasis (P = 0.038; **Table 1**). Kaplan–Meier analysis revealed that the overall survival (OS) rate of patients with a higher ERLIN2 expression was lower than those with a low ERLIN2 expression (P = 0.015; **Figure 4A**). In addition, Cox regression analyses demonstrated that ERLIN2 was an independent predictor for LUAD (P = 0.019; **Figure 4B**). Therefore, these results indicated that ERLIN2 overexpression might predict a poor prognosis in LUAD patients.

**Table 1:** Relationship between endoplasmic reticulum lipid raft-associated protein 2 (ERLIN2) expression level and clinicopathological variables and in lung adenocarcinoma patients.

Variables	ERLIN2 expression		P value
	Low n = 155	High n = 129
Sex, male, n(%)	96 (61.9)	79 (61.2)	1
Age, >60 years, n (%)	94 (60.6)	84 (65.1)	0.514
Smoking, n (%)	41 (26.5)	46 (35.7)	0.122
Tumor size, >3cm, n (%)	91 (58.7)	83 (64.3)	0.397
Differentiation, n (%)			0.044
Well	14 (9.0)	25 (19.4)	
Moderate	105 (67.7)	78 (60.5)	
Poorly	36 (23.2)	26 (20.2)	
Lymph node metastasis, n (%)	58 (37.4)	65 (50.4)	0.038
TNM stage, n(%)			0.01
I	79 (51.0)	47 (36.4)	
II	49 (31.6)	40 (31.0)	
III	25 (16.1)	35 (27.1)	
IV	2 (1.3)	7 (5.4)	
Mortality n(%)	75 (48.4)	85 (65.9)	0.004

### Effect of Endoplasmic Reticulum Lipid Raft-Associated Protein 2 (ERLIN2) on Lung Adenocarcinoma (LUAD) Cell Growth *In Vitro*

**Figure 5:**
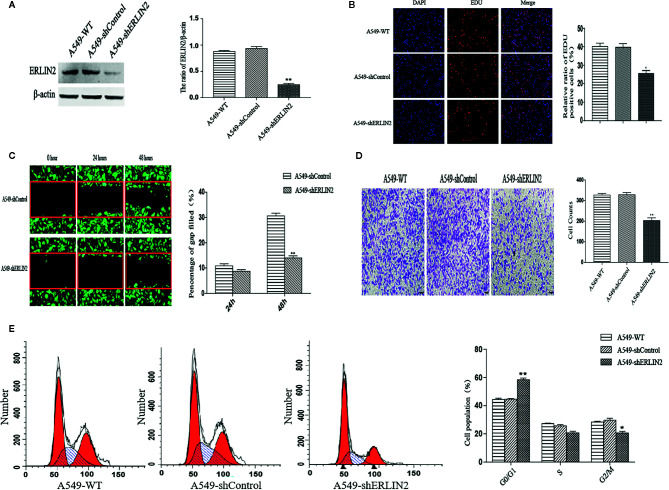
Effects of ERLIN2 on A549 cell proliferation and invasion. **(A)** Interference efficiency of the sh-RNAs in A549 cells. **(B)** Downregulation of ERLIN2 inhibited A549 cell proliferation and viability according to EdU (DAPI represents the number of cells in the field; EdU represents the number of proliferating cells). **(C, D)** Invasion and migration ability of A549 cells was decreased after ERLIN2 knockdown according to the Transwell assay and wound healing assay (*P < 0.05, **P < 0.01). **(E)** Analysis of apoptosis of A549 cells by flow cytometry. After silencing ERLIN2, the cell cycle of G0/G1 phase was blocked in A549 cell.

**Figure 6:**
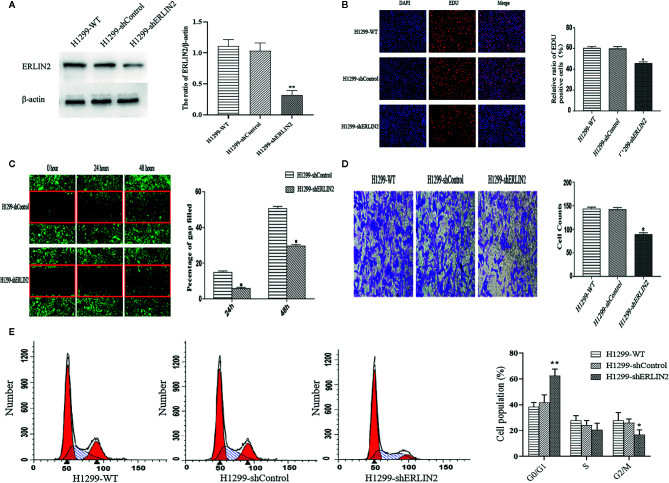
Effects of ERLIN2 on H1299 cell proliferation and invasion. **(A)** Interference efficiency of the sh-RNAs in H1299 cells. **(B)** Downregulation of ERLIN2 inhibited H1299 cell proliferation and viability according to EdU (DAPI represents the number of cells in the field; EdU represents the number of proliferating cells). **(C, D)** Invasion and migration ability of H1299 cells was decreased after ERLIN2 knockdown according to the Transwell assay and wound healing assay (*P < 0.05, **P < 0.01). **(E)** Analysis of apoptosis of H1299 cells by flow cytometry. After silencing ERLIN2, the cell cycle of G0/G1 phase was blocked in H1299 cell.

High levels of ERLIN2 can predict a poor prognosis in LUAD patients, which prompted us to study whether ERLIN2 might be involved in oncogene function. After silencing ERLIN2, expression of ERLIN2 protein decreased in A549 (**Figure 5A**). EdU evaluated how ERLIN2 regulates DNA replication. After silencing ERLIN2, the proliferation of A549 cells was significantly inhibited (**Figure 5B**). Cell healing and invasion experiments showed that silencing the expression of ERLIN2 decreased the migration and invasion abilities of the cells (**Figures 5C, D** **)**. To further study the effect of ERLIN2 on cell growth, flow cytometry was conducted to analyze the cell cycle. Our results indicated that ERLIN2 depletion blocked the cell cycle at the G0/G1 stage in the treated LUAD cells (**Figure 5E**). The same results were shown in H1299 (**Figure 6**).

### Identification of Endoplasmic Reticulum Lipid Raft-Associated Protein 2 (ERLIN2)-Related Protein-Coding Genes in Lung Adenocarcinoma (LUAD)

**Figure 7:**
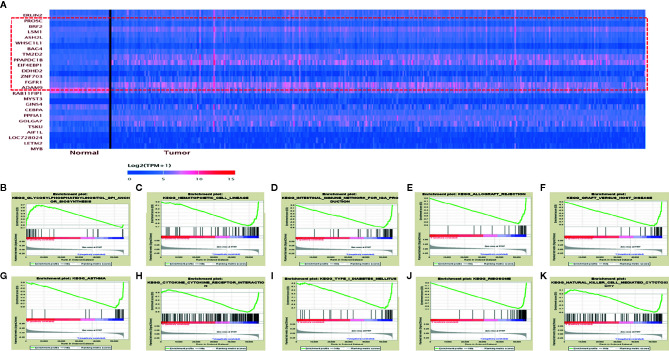
ERLIN2-related protein-coding genes and GSEA pathways in LUAD. **(A)** ERLIN2-related protein-coding genes in LUAD based on TCGA database (Pearson correlation > 0.50). **(B–K)** GSEA pathways using single-gene method of ERLIN2. **(B)** Up-regulated gene sets in the group of high ERLIN2 expression. Glycosylphosphatidylinositol GPI anchor biosynthesis. **(C–K)** Down-regulated gene sets in the group of low ERLIN2 expression. **(C)** Hematopoietic cell lineage. **(D)** Intestinal immune network for IgA production. **(E)** Allograft rejection. **(F)** Graft *versus* host disease. **(G)** Asthma. **(H)** Cytokine–cytokine receptor interaction. **(I)** Type I diabetes mellitus. **(J)** Ribosome. **(K)** Natural killer cell mediated cytotoxicity.

To explore the potential molecular regulatory mechanisms of ERLIN2 in LUAD tumorigenesis, we found protein-coding genes closely related to the expression level of ERLIN2. A two-sided Pearson correlation coefficient analysis and z-test were performed using R based on gene expression data extracted from TCGA. |Pearson correlations| >0.50 and z-test p <0.001 were used as cutoff criteria. Ultimately, 13 protein-coding genes were identified as ERLIN2-related genes (**Table 2**, **Figure 7A**).

**Table 2:** ERLIN2-related protein-coding genes in lung adenocarcinoma (LUAD) based on TCGA database.

Pearson Correlation > 0.50	PROSC; BRF2; LSM1; ASH2L; WHSC1L1; BAG4; TM2D2; PPAPDC1B; EIF4EBP1; DDHD2; ZNF703; FGFR1; ADAM9
Pearson Correlation < −0.50	None

### Gene-Set Enrichment Analysis of Interrelated Pathways

KEGG pathway analyses were conducted to explore the potential biological functions of ERLIN2. GSEA revealed significant differences (FDR < 0.050, p-value < 0.050) in enrichment of the KEGG pathways in samples with high levels of ERLIN2. As shown in **Table 3** and **Figures 7B–K**, KEGG pathway analysis showed the following 10 pathways that had the strongest correlation with ERLIN2 expression: glycosylphosphatidylinositol (GPI) anchor biosynthesis, hematopoietic cell lineage, intestinal immune network for immunoglobulin A (IgA) production, allograft rejection, graft versus host disease, asthma, cytokine receptor interaction, type I diabetes mellitus, ribosome, and natural killer cell (NKC)-mediated cytotoxicity.

**Table 3:** Signaling pathways most significantly correlated with ERLIN2 expression based on their normalized enrichment score (NES) and p-value.

KEGG NAME	ES	NES	NOM p-val	FDR q-val
GLYCOSYLPHOSPHATIDYLINOSITOL _GPI_ANCHOR_BIOSYNTHESIS	0.691	2.068	0	0.026
HEMATOPOIETIC_CELL_LINEAGE	−0.671	−2.169	0.004	0.014
INTESTINAL_IMMUNE _NETWORK_FOR_IGA_PRODUCTION	−0.801	−2.161	0	0.008
ALLOGRAFT_REJECTION	−0.858	−2.091	0.002	0.009
GRAFT_*VERSUS*_HOST_DISEASE	−0.838	−2.088	0.006	0.007
ASTHMA	−0.836	−2.042	0.004	0.008
CYTOKINE_CYTOKINE_ RECEPTOR_INTERACTION	−0.509	−1.956	0.004	0.017
KTYPE_I_DIABETES_MELLITUS	−0.714	−1.898	0.013	0.024
RIBOSOME	−0.873	−1.831	0.006	0.037
NATURAL_KILLER_CELL_ MEDIATED_CYTOTOXICITY	−0.492	−1.829	0.023	0.035

### Association Between Endoplasmic Reticulum Lipid Raft-Associated Protein 2 (ERLIN2) Expression and Tumor Infiltrating Immune Cells (TIIC) Composition

**Figure 8:**
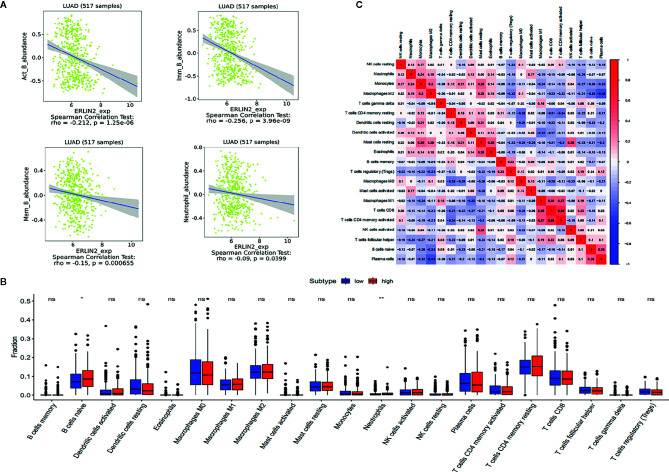
The results of the relative ratios of TIIC that were obtained using the CIBERSORT algorithm and TISIDB. **(A)** Correlations between ERLN2 expression and immune infiltration levels in TISIDB. **(B)** The ratio of 21 immune cells in LUAD tissues in the ERLIN2 high and low expression groups. **(C)** Correlation degree matrix of the relative proportion of immune cells in the microenvironment of LUAD.

As shown in **Figure 8A**, the immune cells that have a significant correlation with ERLIN2 expression were active B cells, memory B cells, immature B cells, and neutrophils. According to the median expression value of ERLIN2, 338 LUAD tumor tissues were downloaded from the TCGA database and divided into high- and low-expression groups comprising169 high- and 169 low-expression groups that met the screening criteria. CIBERSORT was used to explore gene expression profiles of the downloaded samples to determine the levels of 21 types of immune cells. The CIBERSORT algorithm applied to the 21 immune cell subtypes helped to assess the differences in their expression levels in the high- and low-expression groups. Naive B cells and neutrophil cells have difference between the high and low expressions of ERLIN2 (**Figure 8B**). As shown in **Figure 8C**, the correlation heatmap reﬂects a higher correlation within the proportions of different TIIC subgroups. We analyzed the relationship between ERLIN2 expression of TIICs and cell surface markers through the “correlation” module of the Gene Expression Profiling Interactive Analysis tool. This study shows that the immune cells affected by ERLIN2 gene expression were CD8A of CD8+T cells, CD2 and CD3E of T cells (general), CD19 and CD79A of B cells, CCR7 of neutrophils, T-bet, STAT4 of Th1, STAT6 of Th2, BCL-6 of Tfh, STAT3 and IL17A of Th17, CTLA4 of T cell exhaustion, and TPSB2 of mast cells (**Table 4**). We used the Spearman correlation coefficient to evaluate the correlation. The results of B cell and neutrophil markers were similar to those obtained from TISIDB and CIBERSORT.

**Table 4:** A correlation analysis was performed between the gene markers expressed in immune cells and the expression of ERLIN2 using the “Correlation” module of GEPIA.

Description	Gene markers	LUAD			
		Tumor		Normal	
		R	P	R	P
**CD8+ T cell**	CD8A	−0.12	0.011	0.031	0.82
	CD8B	−0.052	0.25	−0.13	0.31
**T cell (general)**	CD2	−0.17	0.00017	−0.019	0.89
	CD3E	−0.18	7.6e-05	0.017	0.9
**B cell**	CD19	−0.17	0.00022	−0.025	0.85
	CD79A	−0.16	0.00048	−0.095	0.47
**Natural killer cell**	KIR2DL1	−0.033	0.46	0.11	0.4
	KIR2DL3	0.0039	0.93	0.15	0.26
	KIR2DL4	−0.039	0.39	−0.16	0.24
	KIR3DL1	0.0022	0.96	0.11	0.41
	KIR3DL2	−0.029	0.53	0.018	0.89
	KIR3DL3	−0.00058	0.9	0.038	0.77
	CD56	0.17	0.00019	0.47	0.00017
**Neutrophils**	CD66b	0.087	0.057	0.062	0.64
	CD11b	0.0014	0.98	−0.027	0.84
	CCR7	−0.15	0.00099	−0.088	0.51
**Th1**	T-bet	−0.12	0.011	0.14	0.28
	STAT4	−0.094	0.038	0.073	0.58
	TNF	−0.053	0.25	−0.055	0.68
**Th2**	GATA3	−0.022	0.63	0.22	0.097
	STAT6	0.19	4.1e-05	0.4	0.0018
	STAT5A	−0.012	0.79	0.086	0.52
	IL13	−0.087	0.057	0.059	0.65
**Tfh**	BCL6	0.17	0.00021	0.34	0.0091
**Th17**	STAT3	0.42	6.6e-22	0.46	3e-04
	IL17A	−0.12	0.008	0.18	0.18
**T cell exhaustion**	LAG3	−0.089	0.05	0.21	0.11
	CTLA4	−0.14	0.0021	0.17	0.2
	TIM-3	−0.046	0.31	−0.41	0.0012
**Mast cells**	TPSB2	−0.13	0.0046	0.16	0.21
	TPSAB1	−0.089	0.05	0.29	0.027
	CPA3	0.048	0.29	0.25	0.057
	MS4A2	0.17	0.063	0.36	0.0052
	HDC	−0.037	0.42	0.18	0.16

## Discussion

Although significant progress has been made in the diagnosis and treatment of LUAD, the 5-year survival of LUAD patients is still poor. Therefore, it is essential to elucidate the molecular mechanisms of LUAD development and identify new prognostic markers and therapeutic targets for LUAD. Previous studies have reported that some proteins including HMGA1, IDH1, CEA and CYFRA play a role in the development of lung adenocarcinoma (16). In the present study, we revealed for the first time that overexpression of ERLIN2 may help to predict a poor prognosis in LUAD Patients. First, the analysis of TCGA RNA-seq data using the TIMER database showed that ERLIN2 mRNA expression was significantly higher in LUAD and some other cancers. We then compared ERLIN2 expression in LUAD tissues with that in adjacent normal tissues using Western blotting, qPCR, and IHC. Our results showed that ERLIN2 was markedly upregulated in LUAD tissues compared with that in normal tissues. Our results also indicated that the expression levels of ERLIN2 were correlated with histological grade, TNM stage, and lymph node metastasis in LUAD patients. In addition, patients with high ERLIN2 expression levels demonstrated a shorter OS time than those with low ERLIN2 expression levels; therefore, these results suggest that ERLIN2 is an oncogene.

ERLIN2 has been reported to be overexpressed in human breast cancer and promotes cancer cell proliferation (8, 17–19). Recent studies have found that the down-regulation of ERLIN2 gene increases cell apoptosis and inhibits the proliferation, invasion and migration of breast cancer cells, which may be related to PI3K/AKT signaling pathway (20).To evaluate the carcinogenic effect of ERLIN2 in LUAD, we knocked down ERLIN2 in A549 and H1299 cells. ERLIN2 knockdown markedly inhibited the growth rate of the tumor cells and reduced other malignant tumor cell behaviors, such as clonogenic survival, colonies counting, and capability for DNA replication. These results suggest that ERLIN2 can promote cell proliferation in LUAD. The unlimited proliferation of cancer cells results in a disorder of the cell cycle (21). Compared with that in the control group, ERLIN2 knockdown arrested the G0/G1 phase in LUAD cells, which indicated that it can also promote LUAD.

Accordingly, we identified the ERLIN2-related protein-coding genes to investigate the potential biological process in LUAD. Based on the TCGA database, we found the following 13 ERLIN2-related protein-coding genes in LUAD: PROSC, BRF2, LSM1, ASH2L, WHSC1L1, BAG4, TM2D2, PPAPDC1B, EIF4EBP1, DDHD2, ZNF703, FGFR1, and ADAM9. To further investigate the functions of ERLIN2 in LUAD, we conducted GSEA using TCGA data, which showed that GPI anchor biosynthesis, hematopoietic cell lineage, intestinal immune network for IgA production, allograft rejection, graft versus host disease, asthma, cytokine receptor interaction, type I diabetes mellitus, ribosome, and NKC-mediated cytotoxicity in KEGG are differentially enriched in the ERLIN2 high-expression phenotype.

Tumor immunotherapy has developed rapidly in recent years, and there is growing recognition of the role of the immune system in the development of cancer (22, 23). Some studies have shown that immune cell infiltration has an influence on survival in lung cancer (24, 25). In our study, CIBERSORT analysis suggested that the expression levels of ERLIN2 had a significant effect on the infiltration levels of naive B cells and neutrophils in the LUAD tumor microenvironment. Similarly, the correlation between B cells, neutrophils, and ERLIN2 expression using TISIDB was the same as that from the CIBERSORT analysis. More and more attention has been paid to the role of tumor-infiltrating B cells in the tumor microenvironment. Most studies have shown that the infiltration of B cells, especially naive B cells, memory B cells, and plasma cells, in non-small cell lung cancer is associated with a good prognosis (26). Meanwhile, neutrophils are potential targets for immunotherapy (27, 28). Previous studies have shown that neutrophil autophagy is closely related to neutrophil immune activity, cytokine secretion, and extracellular trap formation (29, 30); therefore, the signaling pathway of the cytokine–receptor interaction found in our study may be related to neutrophil infiltration. In addition, we used the correlation module in GEPIA to analyze the gene markers in immune cells and the expression of ERLIN2. The correlation between ERLIN2 and surface marker expression in immune cells was basically the same. These results suggest that ERLIN2 may be associated with the immune microenvironment in LUAD.

ERLIN2 is an independent prognostic factor of LUAD, and its high expression suggests a poor prognosis. In addition, ERLIN2 expression significantly correlates with several tumor-infiltrating immune cells, particularly naive B cells and neutrophils. These findings suggest that ERLIN2 can be used as a prognostic biomarker for determining a prognosis and immune infiltration in LUAD patients.

## Data Availability Statement

The datasets presented in this article are not readily available. Requests to access the datasets should be directed to ntdxbiantingting@sina.com.

## Ethics Statement

The studies involving human participants were reviewed and approved by the Ethics Committee of the Affiliated Hospital of Nantong University. Written informed consent for participation was not required for this study in accordance with the national legislation and the institutional requirements.

## Author Contributions

YL and PX conceived the project and wrote the manuscript. DJ conceived the project and participated in the data analysis. JL and JZ collected the data. TB participated in the discussion and language editing. JS reviewed the manuscript. All authors contributed to the article and approved the submitted version.

## Funding

This study was funded by grants from the Key Scientific and Technological Projects in Nantong City, Jiangsu, China (MS22019015), Nantong Municipal Science and Technology Project (No. MSZ19164), Jiangsu Post-doctoral Foundation Research Project, China (No. 2019Z142), Key Talents of Medical Science in Jiangsu Province, China (No. QNRC2016682), and Nantong University Clinical Medicine Special Clinical Basic Research Youth Project (No. 2019JQ001).

## Conflict of Interest

The authors declare that the research was conducted in the absence of any commercial or financial relationships that could be construed as a potential conflict of interest.

## Supplementary Material

Supplementary Figure 1 High or low expression of ERLIN2 in different human cancer tissues compared with normal tissues using the TCGA database.
