# Supplementary figures and images for: Molecular and Immune Characteristics for Lung Adenocarcinoma Patients With ERLIN2 Overexpression
Source: Front Immunol. 2020 Dec 7;11:568440. doi: 10.3389/fimmu.2020.568440 (PMC7793841; doi:10.3389/fimmu.2020.568440)

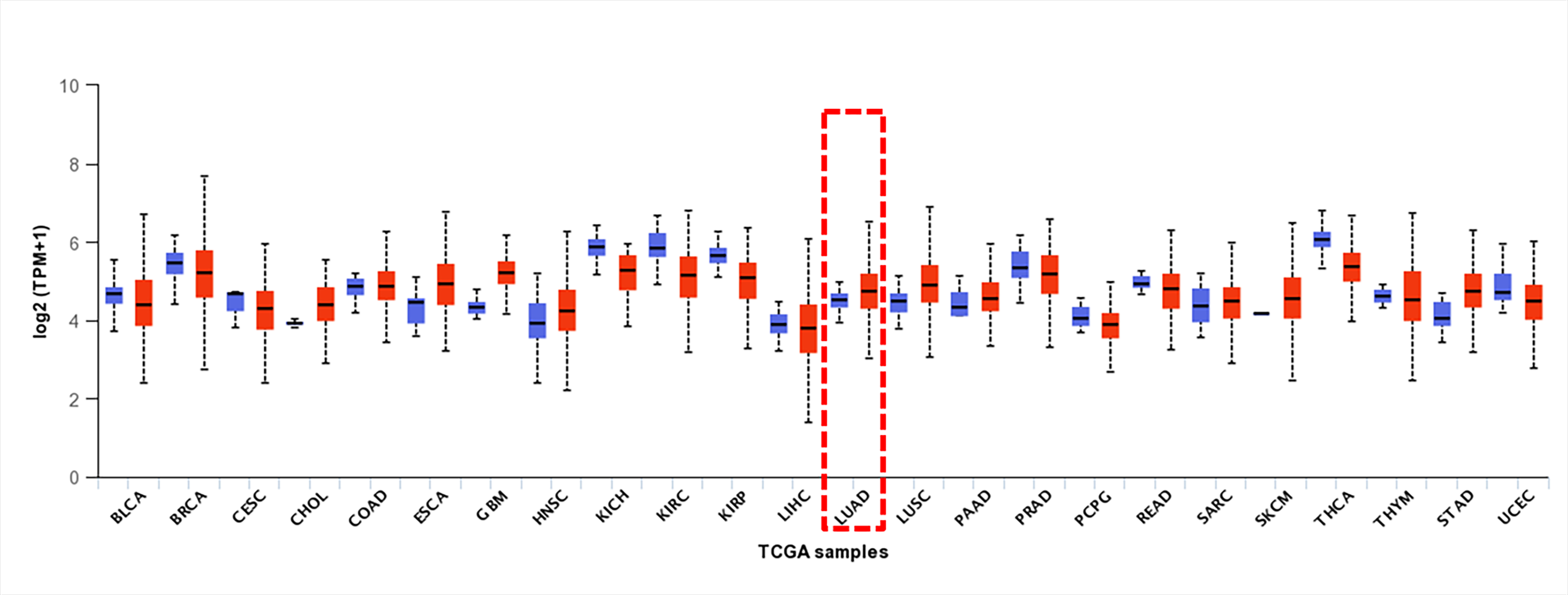

Supplement: Supplementary Figure 1 — High or low expression of ERLIN2 in different human cancer tissues compared with normal tissues using the TCGA database. [file Image_1.tif]
